# Tumor Immune Microenvironment in Lymphoma: Focus on Epigenetics

**DOI:** 10.3390/cancers14061469

**Published:** 2022-03-13

**Authors:** Daniel J. García-Domínguez, Lourdes Hontecillas-Prieto, Natalia Palazón-Carrión, Carlos Jiménez-Cortegana, Víctor Sánchez-Margalet, Luis de la Cruz-Merino

**Affiliations:** 1 Clinical Laboratory, Department of Medical Biochemistry and Molecular Biology, School of Medicine, University of Seville, Virgen Macarena University Hospital, 41009 Seville, Spain; cjcortegana@gmail.com (C.J.-C.); margalet@us.es (V.S.-M.); 2 Oncology Service, Virgen Macarena University Hospital, Department of Medicines, School of Medicine, University of Seville, 41009 Seville, Spain; npalazoncarrion@gmail.com (N.P.-C.); ldelacruzmerino@gmail.com (L.d.l.C.-M.); 3 Department of Radiation Oncology, Weill Cornell Medical College, New York, NY 10065, USA

**Keywords:** lymphoma, epigenetic, tumor microenvironment, immune cells

## Abstract

**Simple Summary:**

Lymphoma and other cancers have been studied by mainly focusing on malignant cells. However, research findings about the role of the tumor microenvironment in the progression and immunosuppression of cancer, particularly in lymphomas, have increased considerably in recent years, allowing for a better understanding of the disease. Consequently, epigenetic mechanisms that are implicated in the interplay between tumor cells and the different components around them, promoting the survival and progression of the tumor, have been described. This review tries to summarize the complex interplay between lymphoma tumor cells and immune cells as well as the epigenetic alterations that result from this cross-talk, aiming at contributing towards underlining the value of epigenetic modifications as new biomarkers and the use of epigenetic drugs as an interesting therapeutic option.

**Abstract:**

Lymphoma is a neoplasm arising from B or T lymphocytes or natural killer cells characterized by clonal lymphoproliferation. This tumor comprises a diverse and heterogeneous group of malignancies with distinct clinical, histopathological, and molecular characteristics. Despite advances in lymphoma treatment, clinical outcomes of patients with relapsed or refractory disease remain poor. Thus, a deeper understanding of molecular pathogenesis and tumor progression of lymphoma is required. Epigenetic alterations contribute to cancer initiation, progression, and drug resistance. In fact, over the past decade, dysregulation of epigenetic mechanisms has been identified in lymphomas, and the knowledge of the epigenetic aberrations has led to the emergence of the promising epigenetic therapy field in lymphoma tumors. However, epigenetic aberrations in lymphoma not only have been found in tumor cells, but also in cells from the tumor microenvironment, such as immune cells. Whereas the epigenetic dysregulation in lymphoma cells is being intensively investigated, there are limited studies regarding the epigenetic mechanisms that affect the functions of immune cells from the tumor microenvironment in lymphoma. Therefore, this review tries to provide a general overview of epigenetic alterations that affect both lymphoma cells and infiltrating immune cells within the tumor, as well as the epigenetic cross-talk between them.

## 1. Introduction

Cancer is a heterogeneous group of diseases that develop from normal cells. The successive abnormalities and changes in these normal cells drive their progressive transformation into malignant cells [1].

Although transformational events were traditionally associated with genetic changes [1], we currently know that cancer is caused by both genetic and epigenetic alterations. Each change provides advantageous properties to cancer cells, such as uncontrolled cell proliferation, resistance to cell death, evasion of apoptosis, invasiveness, and metastatic potential [2,3]. Thus, the interest in the epigenetic field has increased over the last decade in a wide variety of tumors. Given the importance of epigenetics in affecting cell function by providing disruptive properties, a better understanding of how epigenetic modifications regulate both tumor cells and the tumor microenvironment (TME) is mandatory. Tumors are a group of malignant cells that constantly interact with the TME, which contains different cell types, such as immune cells [4]. Lymphoma cells have been described to interact with the microenvironment to promote immune evasion [5]. In fact, the reciprocal interaction between lymphoma cells and immune cells (such as T and B cells) plays a key role in cancer initiation and progression [6,7]. In addition, immune cell function in the TME is affected by epigenetic alterations, which contributes to a favorable environment for tumor growth. As a result, tumor cells control immune cell functions through complex signaling networks, in which epigenetics plays an essential role.

Summarizing, the epigenetic cross-talk between tumor cells and immune cells promotes tumor growth and immune escape of malignant cells [8,9], as well as a deficient response to therapy [10] both in solid and hematological cancers, including (but not limited to) lymphomas. Therefore, the underlying epigenetic mechanisms that govern both tumor and immune cells can be used as a strategy to disrupt this interaction and contribute to developing therapeutic strategies against cancer.

## 2. Lymphoma: An Overview

Lymphoma represents a diverse and heterogeneous group of tumors and is among the ten most prevalent types of cancers worldwide [11]. This neoplasm arises from a clonal proliferation of lymphocytes. Specifically, lymphoma arises from B cell, T cell, or natural killer (NK) cell subsets at different stages of maturation [12,13].

Due to the diversity of lymphoid tumors, the first international classification of lymphoma malignancies was published in 2001 by the World Health Organization (WHO) [14]. Then, the classification was updated in 2008 and 2017 [14] to include important advances that had an impact on the diagnostic approach as well as on new therapeutic strategies for lymphoid neoplasms. The latest revision recognized more than 80 lymphoma subtypes classified into three main categories: B cell, T cell and NK cell neoplasms, and Hodgkin lymphomas. However, lymphomas have traditionally been classified as: (1) Hodgkin lymphoma (HL), which is characterized by the presence of Reed–Sternberg cells and accounts for 10–15% of cases, and (2) Non-Hodgkin lymphoma (NHL), which includes B cell NHLs, T cell NHLs (T NHLs), and natural killer (NK)/-cell NHLs and accounts for 80–85% of cases.

Establishing the lymphoma diagnosis is essential to evaluate the clinical and histopathological features with molecular and immunologic studies [14], which, altogether, will provide better knowledge about both the severity and prognosis of the disease. Thus, for therapeutic purposes, the general practice is to treat lymphoma patients based on limited/indolent (low grade) or advanced/aggressive (high grade) disease [15]. Although indolent lymphomas have favorable prognoses, they are not usually curable at advanced stages. For this reason, relapsed and/or refractory patients have poor prognosis.

There are multiple approved chemotherapy schedules in lymphoproliferative syndromes. Their use will depend mainly on the type of lymphoma, stage, disease situation (previously untreated or relapsed/refractory lymphoma), and characteristics of the patient (medically fit/unfit, comorbidities, age).

For untreated diffuse large B cell lymphoma (DLBCL), the most used schedule includes systemic chemotherapy (doxorubicin, cyclophosphamide, vincristine, and prednisone) plus immunotherapy with the recombinant anti-CD20 antibody rituximab (R-CHOP-21) given every 21 days, with estimated rates of progression-free survival (PFS) and overall survival (OS) of 70% and 75% for the advanced stage and rates of PFS and OS of 90% and 95% for the limited stage [16,17]. Although other chemoimmunotherapy regimens and schedules (dose-dense R-CHOP-14 or ACVBP—doxorubicin, cyclophosphamide, vindesine, bleomycin, prednisone followed by consolidation with methotrexate, etoposide, ifosfamide, cytarabine) have been used to treat DLBCL, none has a more favorable balance of outcomes and toxicity than R-CHOP [18,19]. For patients with advanced stage double-triple hit DLBCL (lymphomas with rearrangements of MYC and BCL2 and/or BCL6), acceptable intensive chemotherapy regimens include: dose-adjusted etoposide, doxorubicin, vincristine, cyclophosphamide, and prednisone plus rituximab (da-EPOCH-R), rituximab plus hyperfractionated cyclophosphamide, vincristine, doxorubicin and dexamethasone alternating with methotrexate and cytarabine (R-HyperCVAD/MA), rituximab plus cyclophosphamide, vincristine, doxorubicin, and high-dose methotrexate alternating with ifosfamide, etoposide, and cytarabine (R-CODOX-M/IVAC) given the worse prognosis of these patients. When compared with R-CHOP, more intensive front-line therapy (da-EPOCH-R, R-HyperCVAD/MA, R-CODOX-M/IVAC) was associated with superior three-year PFS (88 vs. 56%) [20]. Salvage chemotherapy (R-GDP, rituximab, gemcitabine, dexamethasone; R-ICE, rituximab, ifosfamide, carboplatin; R-DHAP, rituximab, dexamethasone, high dose cytarabine, cisplatin; R-GEMOX, rituximab, gemcitabine, oxaliplatin) followed by autologous hematopoietic cell transplantation (HCT) has long been the standard care in medically-fit patients with relapsed/refractory DLBCL (R/R DLBCL). It is associated with long-term survival in approximately half of patients with first relapse of DLBCL, and achieves superior OS (53 vs. 32%), overall response rate (ORR) (84 vs. 44%), and PFS (46 vs. 12%) compared with salvage therapy alone [21]. For patients unsuitable for autologous HCT, salvage treatments with rituximab combined with chemotherapy have a median OS and PFS of 10 and 6 months, respectively [22]. Other regimens in R/R DLBCL include polatuzumab–rituximab–bendamustine (median OS and PFS of 12 and 9 months) [23], tafasitamab–lenalidomide (median OS and PFS of 33 and 11 months) [24], and CAR T-cell therapies (ORR of 50–85% and CR of 47%) [25,26].

A treatment option for follicular, indolent, or mantle cell lymphoma is bendamustine plus rituximab (BR). Trials suggest similar efficacy and fewer side effects when compared with R-CHOP (median PFS 69.5 vs. 31.2 months; no difference in OS). ABVD (doxorubicin, bleomycin, vinblastine, and dacarbazine), the standard initial chemotherapy for patients with Hodgkin lymphoma (HL), shows OS rates at 5 and 10 years of approximately 85% and 55%, respectively, and CR rate of 80% in advanced stage [27].

Bleomycin, etoposide, doxorubicin, cyclophosphamide, vincristine, procarbazine, and prednisone (BEACOPP) and variants of BEACOPP have shown advantages in FPS (85% with BEACOPP and 73% with ABVD, *p* = 0.004), but not OS (89% vs. 84%, respectively, *p* = 0.39), when compared with ABVD in several randomized trials in HL; additionally, severe adverse events occur more frequently in the BEACOPP than in the ABVD schedule [28,29]. Short-term outcomes with BV + AVD (brentuximab vedotin, an anti-CD30 antibody-drug conjugate, doxorubicin, vinblastine, and dacarbazine) are at least as favorable as ABVD, but cause less pulmonary toxicity, so this treatment is an acceptable alternative to ABVD for patients at higher risk for pulmonary toxicity and HL [30]. Patients with relapse/refractory HL (R/R HL) are generally treated with targeted chemotherapy BV plus bendamustine or intensive combination chemotherapy (ICE, DHAP, or gemcitabine-containing regimens) and, if a response is demonstrated, start of autologous HCT, if eligible, with results in PFS rates from 30% to 50% [31].

Chemotherapy has improved the outcomes of lymphoma patients over the immunophenotypic subgroups and is the standard treatment for those patients, together with other treatments such as radiation, stem cell transplantation, targeted therapies, or immunotherapies [32,33,34]. Immunotherapy has become a tool for lymphoma treatment as it restores the anti-tumor response of the patient’s immune system. It is described that Programmed Death 1 (PD-1) ligands, PD-L1 and PD-L2, are overexpressed by Hodgkin Reed–Sternberg (HRS) cells in classic HL (cHL), leading to evasion of immune surveillance. Therefore, anti-PD-1 antibodies such as nivolumab and pembrolizumab are used in lymphoma patients. Prospective phase II studies have reported high response rates with nivolumab and pembrolizumab in R/R cHL, but further study is required to establish the durability of treatment [35,36,37]. Nivolumab is approved for patients with cHL who relapsed or progressed after autologous HCT and post-transplantation brentuximab vedotin. Nivolumab showed 69% OR, including 16% CR and median PFS of 15 months [35]. Pembrolizumab is approved in the United States for patients whose disease is refractory or has relapsed after two or more lines of therapy and in the European Union for patients with progression after autologous HCT and brentuximab vedotin, or who are transplant-ineligible and have failed brentuximab vedotin. Pembrolizumab reported 72% OR, including 28% CR, and 14-month median PFS in heavily pretreated patients with r/r HL [36,37].

Although all these treatments have improved the outcomes of lymphoma patients, it is important to develop and identify novel strategies and therapies that could reduce drug resistance and increase the survival rate by minimizing adverse events. In fact, preclinical data and clinical trials are showing epigenetic agents as promising and potential therapeutic tools.

## 3. Tumor Microenvironment: Interaction of Immune and Lymphoma Tumor Cells

Malignant cells need a dynamic and mutual communication with the components around the tumor to evade immune responses and progress. These components are a heterogeneous mix of immune cells, stromal cells, blood and lymphatic vessels, secreted factors, and extracellular matrix [38,39] which shape the TME. Thus, this interaction plays a pivotal role in supporting the survival and growth of the tumor, the invasion and metastasis, as well as the drug resistance and immune evasion [40,41]. As a result, the composition of the TME has been described to vary according to different cancer types and can predict the patients’ outcome in many cancers, including lymphomas [42]. For example, the TME constitutes more than 80% of the tumor mass in HL and several T-cell lymphoma subtypes, whereas it constitutes approximately 50% in indolent B-cell lymphomas or it is lower in aggressive lymphomas, DLBCL [42].

Immune cells are able to infiltrate tumors by promoting both pro- and anti-tumorigenic functions (Table 1). This dual relationship between immune cells and tumor cells depends on the context. Therefore, macrophages, dendritic cells (DCs), eosinophils, or tumor-infiltrated lymphocytes (TILs; T cells, B cells, and NK cells) have been demonstrated to be relevant for tumor control [43], whereas myeloid-derived suppressor cells (MDSCs), mast cells (MCs), regulatory T cells (Tregs), and type 2-polarized tumor-associated macrophages (TAMs) are important in immunosuppressing action [44].

Given the role of the immune cells in the behavior of the tumor, a deeper knowledge of interactions between them would be relevant to better understand the pathogenesis and prognosis of lymphomas as well as new therapeutic targets. For this reason, the interactions between lymphoma cells and surrounding immune cells are now being studied in much more detail. This section describes the interactions between lymphoma cells and some immune cells of the TME.

**Lymphoid cells:** Lymphoid cells or lymphocytes are a type of white blood cell that develops from lymphoid progenitor cells that include T cells, B cells, and NK cells. These three types of lymphocytes are commonly found in the TME in lymphomas.

(a) T cells are essential for both immune surveillance and disease progression. Therefore, they are being extensively studied in all types of cancers, including lymphoma subtypes [45,46]. The T cell compartment includes a variety of cell subpopulations with different immune functions: CD4+ T-helper cells (Ths), CD4+/FOXP3+ Tregs, and CD8+ cytotoxic T cells (CTLs). Additionally, there are two types of Th cells, which are Th1 and Th2 cells. Th1 cells produce interleukin (IL)-2, gamma-interferon (IFN-γ) and tumor necrosis factor-beta (TNF-beta), activating CTLs, antigen-presenting cells (APCs), and NK cells [47]. In the meantime, Th2 cells express IL-4, IL-5, IL-6, and IL-10 and support tumor cell growth through CD40–CD40 ligand (CD40L) interactions in HL and Helicobacter-induced MALT lymphomagenesis [6,48]. Accordingly, lymphoma tumor cells can increase IL-10 secretion to produce CD40L by T cells [49]. In fact, IL-10RA or IL-10RB gene amplifications are described in DLBCL, promoting the tumor’s survival [50,51]. In follicular lymphoma (FL), Th cells are predominant and secrete IL-2, IL-4, IFN-γ, and CD40L [52].

It has been described that T cells surround but do not destroy tumor cells in vitro in HL, probably because T cells are less responsive and are attenuated in this disease [53]. In line with this notion, lymphoma tumor cells induce the expression of programmed cell PD-L1, and both CD4+ and CD8+ T-cells express PD-1, thus, the PD-1/PD-L1/2 interaction could suppress the activity of T cells and promote their exhaustion [54]. As a consequence, lymphoma tumor cells drive T cells to apoptosis or promote their differentiation towards Th2 via PD-L1 expression [55], which also enables an immunosuppressive environment in HL through the interaction with PD-1 from macrophages [56].

Tregs are abundant in B-cell NHL, such as FL or DLBCL, showing an immunosuppressive capacity [57,58]. However, the relevance of tumor infiltrating Tregs on disease outcome is unclear. In FL, FOXP3-positive Tregs have been considered as a negative prognostic factor [59], either alone or in cooperation with M2 macrophages from the TME. Nevertheless, FOXP3+ Tregs have also been associated with better outcomes in HL and other lymphoma subtypes [60], despite the high PD-1+ T cells [61] and CD68+ macrophages [62] that have been found. In DLBCL, high levels of Tim-3+Foxp3+Tregs within the TME have been considered as a prognostic factor for poor survival [58], whereas the high intratumor infiltration of CD25+FOXP3+ Tregs was correlated with a favorable prognosis in a meta-analysis [63].

Given the controversy in the role of different Treg subtypes in the prognosis and their capacity to express co-inhibitory and co-stimulatory markers, a recent study performed an in-depth characterization of Treg heterogeneity in NHL. Three different Treg subsets have been identified up to now, revealing the heterogeneity within the Treg compartment. The first one displayed a low expression of PD-1, OX40, and CTLA-4 that corresponded to peripheral blood Tregs. The second subset had high levels of PD-1, OX40, ICOS, TIGIT, CTLA-4 and CD28, CD69, and CD95/Fas (activation markers), and the third subset had a lack of FOXP3 and a high expression of LAG3, CTLA-4, IL-10, CD38, and KLRB1 (immunosuppression-associated genes) [57].

CD8+ cytotoxic T cells (CTLs) play an important role in infectious and malignant diseases. Thus, the anti-tumor effect of CTLs can be reversed depending on the type of lymphoma [64,65]. In this sense, the progression of Epstein–Barr virus (EBV)+ HL [66] or B-cell NHL [67] is suppressed by CTLs, whereas CTLs have also been associated with progression of CD8+ lymphomas, such as nodal cytotoxic T-cell lymphoma and cutaneous T-cell lymphoma (CTCL), towards their malignant transformation [68,69].

(b) B cells are the main humoral immune cells that play a role in antitumor immunity. In indolent lymphomas, non-malignant B cells are frequently encountered in the TME, but their role remains poorly understood [38,45]. Regarding their relationship with lymphoma tumor cells, normal and activated B cells from the germinal center express CD95 (also called FAS) to induce apoptosis. However, the loss of CD95 has been described in many lymphomas, including FL, MALT, and DLCBL [70,71,72]. Additionally, the expression of CD47 has been found in lower levels on normal B cells than in malignant B cells [73] in NHL, which could contribute to immune evasion. Finally, in mature B-cell lymphoma, B cells allow the progression of the disease inducing anergy in CD4+ T cells by CTLA-4 up-regulation [7].

(c) NK cells play a role in the response against infection and tumor cells, so the activation and detection of NK cells in the lymphoma tumor confer a favorable prognosis [74]. Due to the antitumor function of NK cells, lymphomas contribute to the quantitative and functional deficiency of NK cells, which prevents tumor elimination [75]. Regardless of the HL subtype, the concentration of NK cells could be five times lower in affected tissues in comparison with normal tissues [75]. Moreover, a lower number of tumor infiltrating NK cells in HL was associated with poor prognosis and advanced clinical stages [76]. Many factors have been described to contribute in this reduction, such as IL-2 inhibition or the interruption of NKp30 and NKG2D surface receptors [77]. Indeed, a weak HL-derived NK cells cytolysis against the L428 HL cell line was observed by Reiners et al., in contrast to healthy donors where NK cells were able to efficiently eliminate lymphoma tumor cells [77]. This functional deficiency may be related to the significant reduction in NKG2D in NK cells of HL patients (*p* = 0.0001 for healthy versus untreated patients with HL) [77].

**Tumor-associated macrophages (TAMs):** Macrophages act against infection and injury via phagocytosis [78]. Due to their relevant role in TME, these cells are called TAMs, which have two different phenotypes depending on their activation states during tumor progression: M1 macrophages, which have anti-tumor effects because of their cytotoxic actions; and M2 macrophages, which promote tumor activity and support tumor growth [79]. The polarization towards M1 or M2 is influenced by cytokines secreted from both Th1 and Th2 cells, respectively [80].

Specifically in lymphomas, malignant cells were described to be able to induce macrophages to their M2 phenotype in vitro [81], which were associated with poor outcomes [82,83]. Although macrophages have been traditionally characterized by the expression of CD68 (a pan-macrophage marker that recognizes epitopes in a wide variety of tissue macrophages), several studies have revealed that M2 macrophages also express CD163 [84]. Accordingly, CD163+ TAMs have been associated with poor prognosis in many types of cancer, including lymphomas [85,86]. In DLBCL, a high number of CD68+/CD163+ or CD163+ M2 at diagnosis was significantly correlated with unfavorable prognosis [87,88].

In addition to CD68 and CD163 markers, it has also been described that MYC controls the expression of M2 specific genes, and its depletion in MYC+ macrophages inhibited tumor growth in melanoma and fibrosarcoma mouse models [89]. In line with this, a recent study evaluated the relevance of MYC-negative and -positive macrophages in HL patients based on positive CD68 and CD163 markers. The authors subdivided the patients into low, intermediate, and high groups based on the number of macrophages over the total number of CD68+ and CD163+ macrophages, the number of MYC+ macrophages, and the number of MYC−macrophages. The results showed that the worst outcomes were associated with a high number of CD163+/MYC+ cells, whereas the best outcomes were correlated with intermediate levels of CD68+/MYC+ macrophages [80].

**Myeloid derived suppressor cells (MDSCs):** MDSCs, originated from immature myeloid cells, are a group of heterogeneous cells characterized by suppressing immune responses [90]. Currently, MDSCs are generally defined by CD45+ CD3− CD19− CD20− CD56− CD16− HLADR− CD33+ CD11b+ through flow cytometry [91]. Besides, there are two different subpopulations of MDSCs that can either express CD14 or CD15: monocytic MDSCs (M-MDSCs), which express CD14 and may differentiate into monocytes (such as macrophages and dendritic cells), and granulocytic MDSCs (G-MDSCs), which express CD15 and may differentiate into granulocytes (polymorphonuclear leukocytes) [90,91,92]. MDSCs mainly inhibit T cell immune responses by using different mechanisms, such as reactive oxygen species or nitrogen oxide production [93] and Tregs induction, which contributes to tumor dissemination [94]. Through the production of angiogenic factors and proteases, MDSCs also promote angiogenesis [95]. Additionally, MDSCs inhibit the maturation and functions of NK cells and induce the polarization of macrophages into their M2 phenotype by secreting IL-10 and transforming growth factor (TGF)-β [95,96].

Regarding the prognosis in lymphoma, the increased number of MDSCs has been correlated with a more aggressive disease in both HL and NHL [97,98]. Thus, MDSC expansion has been associated with tumor growth and the promotion of a immunosuppressed TME in HL [99]. Moreover, higher levels of MDSCs reduced the response rate in lymphoma patients [99]. Thus, MDSC count was increased compared with healthy controls (12.5% vs. 11%, respectively), but was higher in non-responders (19.7%) [99]. For this reason, several studies support the hypothesis that MDSCs may be promising prognostic biomarkers in lymphomas such as DLBCL [93], and, even more, the results also support their relevance as potential therapeutic targets to overcome immunosuppression [93].

Other studies have also evaluated the relevance of M-MDSCs and G-MDSCs in patients with lymphoma. In B-cell NHL and T-NHL patients, an accumulation of M-MDSCs was described in peripheral blood in comparison with healthy donors [100,101,102], which was correlated with poor survival and advanced lymphoma stage [100,102,103]. It was also noted that M-MDSCs returned to normal levels after clinical tumor remission and T cells were reestablished after removing M-MDSCs [101,102,104,105]. This result has also been described for G-MDSCs, wherein the median percentage of G-MDSCs was higher in patients with HL and B-NHL when compared to healthy donors (2.18 (0.02–70.92) vs. 0.42 (0.04–2.97), *p* < 0.0001). Considering HL, indolent, and aggressive B-cell NHL patients separately, the percentage of G-MDSCs was higher in the second group of patients (1.54 (0.28–26.34), (2.15 (0.02–20.08), and 2.96 (0.25–70.92), respectively, *p* < 0.0001) [98]. Moreover, high G-MDSC levels were found in the duodenum related to enteropathy-associated T cell lymphoma [106].

## 4. Epigenetic

Epigenetic is a term introduced by developmental biologist Conrad Hal Waddington in 1942 in order to explain the dynamic interactions between the developmental environment and the genome [107]. Nowadays, epigenetic is generally accepted and defined as “the study of changes in gene function that are mitotically and/or meiotically heritable and that do not entail a change in DNA sequence” [108]. Epigenetics promotes changes in the chromatin structure, which regulates gene expression. These changes, unlike genetic changes, are reversible and comprise DNA methylation, histone modifications, and non-coding RNA [109]. Although the epigenetic role was first described as an essential mechanism for normal cell function, later, it was found that epigenetic disruptions promoted malignant cellular transformation leading to diverse human diseases, including cancer [110,111,112]. Pediatric tumors, such as Ewing sarcoma, are an example of the relevance of epigenetics in cancer. Indeed, pediatric tumors have been described to have a low frequency of somatic mutations together with hematological tumors [113]. The low frequency of mutations in these tumors does not explain the differences between those patients with different outcomes. Therefore, epigenetics constitutes a promising research area in order to understand the role of the epigenetic alterations in cancer and look for new alternatives to conventional strategies [114,115].

### 4.1. Epigenetic Alterations in Lymphoma

**Figure 1:**
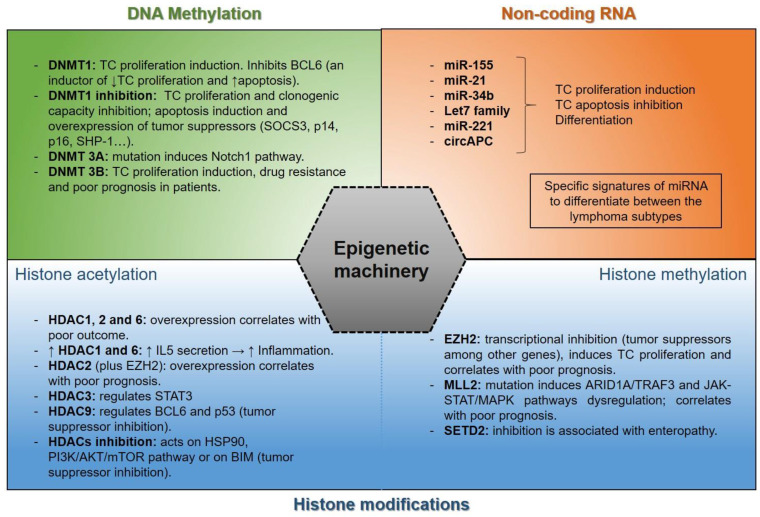
DNA methylation, non-coding RNA, and histone modifications as regulatory epigenetic mechanisms in lymphoma tumor cells.

Increasing advances have allowed researchers to understand the relevance of epigenetic modifications, both in normal hematopoiesis and lymphoid development and in lymphoma. In fact, it has been described that changes in DNA methylation, histone modifications, and non-coding RNA are implicated in the development of B cells and in the maintenance of T-cell identity [116,117,118]. Moreover, it has been described that epigenetic alterations could be important to uncover the response to therapy and the prognosis in lymphoma malignancies [119,120] (Figure 1 and Table 2).

The most common epigenetic alterations in lymphoma are:

**DNA methylation:** The process catalyzed by DNA methyltransferases (DNMTs), in which a methyl group is transferred to the C-5 position of the cytosine ring of DNA [121], is called DNA methylation. This epigenetic mechanism occurs within promoter regions where methylation regulates gene expression by transcription silencing [122]. The DNMT family is comprised of DNMT1, DNMT2, DNMT3A, DNMT3B, and DNMT3L members [123,124]; however, DNMT1, DNMT3A, and DNMT3B are the only DNMTs implicated in lymphopoiesis and B-cell activation at early stages [125,126].

DNMT1 plays a relevant role in many types of lymphomas. In this regard, DNMT1 has been described to be frequently expressed and to promote cell cycle and DNA replication in DLBCL cells [127]. Moreover, the complex form by AID–DNMT1 inhibits BCL6 expression leading to cell apoptosis and the inhibition of tumor growth in DLBCL cell xenograft mice [128]. In T-cell lymphoma, Peters et al. reported that DNMT1 loss impaired tumor cell proliferation, induced apoptosis, and suppressed normal hematopoiesis. Thus, the authors suggested that DNMT1 was implicated in the maintenance of the tumor phenotype in MYC-induced T-cell lymphomas and in the de novo methylation during tumorigenesis [129]. Downregulation of DNMT1 by shRNA inhibited proliferation, cell cycle and clonal formation, as well as induced apoptosis in OCI-Ly10 and Granta-159 lymphoma cell lines. This downregulation significantly upregulated the expression of tumor suppressor genes such as SOCS3, BCL2L10, p16, p14, and SHP-1 [130].

In Burkitt’s lymphoma (BL), DNMT1 is overexpressed [131]. In fact, the *MYC* oncogene promotes overexpression of DNMT1, which results in tumor maintenance and progression in BL [132].

DNMT 3B overexpression plays a role in the progression in lymphomas, being predominantly reported in BL [131]. DNMT 3B is approximately overexpressed in 86% of BL patients, contributing to DNA methylation with DNMT1 [131]. This overexpression is due to the direct binding of the oncoprotein MYC to the DNMT 3B promoter region [132]. In addition, in DLBCL, the overexpression of DNMT 3B has been reported and is associated with worse prognosis, treatment resistance, and progression of the disease [132,133].

Even though DNMT1 and DNMT 3B are the most frequently overexpressed and mutated DNMTs in lymphoma, DNMT 3A has been found to be mutated in nearly 11–20% of patients with T-cell lymphoma [134]. DNMT 3A mutations promote its complete loss of function because of the biallelic mutations observed, which could be important in the development of these neoplasms [135]. This loss of DNMT 3A function is mainly the result of the missense mutations, and also, to a lesser extent, nonsense or frameshift mutations [136,137]. DNMT 3A mutation is correlated with Ten-Eleven Translocation 2 (TET2) mutation in peripheral T-cell lymphoma (PTCL) [134,135]. These two mutations would confer selective advantages through the activation of Notch1 signaling pathway by hypomethylation [138,139], or either by RhoA and/or IDH2 mutations [140,141].

**Histone modifications:** Acetylation, methylation, phosphorylation, and ubiquitination are histone modifications. In lymphomas, acetylation and methylation of histones are the most frequent aberrations.

(a) Histone acetylation: Chromosome structural modification and regulation of gene expression are regulated by the functional balance between histone acetyltransferases (HATs) and histone deacetylases (HDACs), which contribute to acetylation and deacetylation of histones. Thus, an acetyl group is transferred from acetyl-CoA to the lysine residues NH_2_ group in proteins by HATs and is removed by HDACs [142,143].

Aberrant expression of HDACs in lymphomas has been reported to contribute to clinical outcome. In PTCL and DLBCL, HDAC1, HDAC2, and HDAC6 were overexpressed when compared to normal lymphoid tissue. Moreover, HDAC6 overexpression was correlated with favorable outcome in DLBCL patients, whereas it had the opposite effect in PTCL [144]. In line with this, the co-overexpression of EZH2 and HDAC2 in PTCL patients has been associated with a poorer survival rate [145].

Additionally, oncoproteins and non-histone proteins are also able to be modified by HDACs and HATs. For example, HDAC3 regulates the signal transducer and activator of transcription (STAT) 3 in DLBCL [146]. HDAC1 and HDAC6 upregulation promotes an increase in IL-15 secretion, contributing to inflammation in CTCL [147]. HDAC9 overexpression modulates BCL6 activity and the function of the tumor suppressor p53 in B-cell lymphomas [148]. In addition, HDACs’ inhibition can act on chaperone protein HSP90 in B-cell lymphoma [149], on the PI3K/AKT/mTOR pathway in PTCL patients [150], or on BIM (tumor suppressor in lymphomagenesis) through SIN3a/HDAC1/2 co-repressor complex in anaplastic large-cell lymphoma [151].

(b) Histone methylation: Histone methyltransferase enzymes are responsible for transferring methyl groups to lysine and arginine of histones. Mono-, di-, and trimethylation affects gene transcription and promotes lymphoma pathogenesis [152]. Several methyltransferases, such as EZH2, MML2, or SETD2, among others, are associated with lymphoma tumors. SETD2 (SET-domain-containing 2), a methyltransferase responsible for H3 lysine 36 trimethylation (H3K36me3), is mostly silenced in enteropathy-associated T-cell lymphoma (EATL) [153], as well as monomorphic epitheliotropic intestinal T-cell lymphoma (MEITL) [154]. MLL2 (also known as KMT2D) is mutated in angioimmunoblastic T-cell lymphoma (AITL), PTCL not otherwise specified (PTCL-NOS) [155], DLBCL, FL [156], and NK/T-cell lymphoma (NK/TCL) [157,158], where approximately 91% of mutations result in silencing its histone methylation function [159]. The loss of MLL2 functions alters several genes such as ARID1A, TRAF3, or signaling pathways such as JAK-STAT [160] or MAPK. In fact, the combination of chidamide and decitabine improves the KMT2D-PU.1 transcription factor interaction, which inactivates the MAPK pathway [161], constitutively activated in T-cell lymphoma [162]. Likewise, the loss of MLL2 functions influences the poor prognosis in lymphoma patients [157,158,163].

The enhancer of Zeste Homolog 2 (EZH2) catalyzes histone H3 lysine 27 tri-methylation (H3K27me3), which is related to downregulation or suppression of gene transcription [164,165]. EZH2 SET domain is responsible for the transcriptional inhibition mediated by EZH2. A tyrosine deletion/mutation (Y641) at the EZH2 SET domain in DLBCL and FL patients increases H3K27me3 levels repressing tumor suppressor genes and cell differentiation [166]. In different human lymphoma cell lines, the mutation A677G in EZH2 showed analogous effects [166,167]. The relevance of EZH2 is highly reported in several lymphomas. In aggressive B-cell lymphoma, EZH2 is overexpressed in most cases. Additionally, a positive correlation with phosphorylated ERK1/2 (p-ERK1/2) in DLBCL and MYC expression in BL and double-hit lymphoma was observed [168]. EZH2 overexpression was also described in most T-cell lymphomas, such as NK/TCL [169], T-lymphoblastic lymphoma (T-LBL) [170], or adult T-cell leukaemia/lymphoma (ATLL) [171]. This overexpression has been associated with a high proliferation and poor prognosis in PTCL patients [145] and in NKTCL [172].

**Non-coding RNA:** Non-coding RNA are transcripts that do not encode proteins and are classified based on their length and structure in microRNA (miRNA), long noncoding RNA (lncRNA), and circular RNA (circRNA) [173]. In the last years, many studies have reported the non-coding RNA expression profile in lymphomas, especially B-cell NHLs.

In DLBCL, the most common and frequently aggressive NHL, numerous miRNAs with biological functions, such as miRNA-155 or miRNA-21, have been described to be deregulated [174,175,176]. In BL, which presents a translocation of c-Myc and immunoglobulin genes, a deregulation of some miRNAs controlled by c-Myc, such as let-7 family or miR-34b, among others, has been reported [177,178].

Additionally, different studies have focused on searching for the differences among lymphoma subtypes or between subgroups within a lymphoma. A study showed a miRNA signature that distinguishes between two groups of DLBCL, which are germinal center B-cell-like DLBCL (GCB-DLBCL) and activated B-cell-like DLBCL (ABC-DLBCL). This miRNA signature included the miRNAs 155, 21, and 221, which contributed to discriminating between GCB- and non-GCB DLBCL [174]. The miRNA-155 differential expression in ABC-DLBCL was confirmed in other independent studies [174,175,179]. A specific miRNA signature that could differentiate between BL and DLBCL has also been found [180,181]. In FL, 17 miRNAs associated with differentiation, apoptosis, and proliferation were differentially expressed with respect to t(14;18) (q32;q21) positive and negative FL patients [182].

Regarding lncRNA, a comparison between control B cells and DLBCL samples has shown 2632 novel lncRNAs differentially expressed in this disease [183], and 17 lncRNAs were able to make a distinction between GCB- and ABC-DLBCL subgroups [184]. In FL, 189 deregulated lncRNAs have been observed [185]. Additionally, a recent study has described that the lncRNA NKILA is frequently hypermethylated in DLBCL and its silencing promoted an increase in proliferation and a decrease in cell death in vitro by repression of NF-kB signaling in NHL [186].

There are only a few studies on lymphomas that evaluated the relevance of circRNA expression. Dahl M et al. provided a circRNA expression map in many B-cell malignancies. Their results showed circRNA profiles that could distinguish different B-cell malignancies [187]. Moreover, Hu et al. identified many differentially expressed circRNAs comparing three pairs of DLBCL tissues and adjacent tissues [188]. circ-APC (hsa_circ_0127621) was downregulated in DLBCL tissues, cell lines, and plasma, and the in vitro and in vivo studies verified that the proliferation of DLBCL cells and tumor growth was impaired by circ-APC [188].

### 4.2. Epigenetic Regulation of Immune Cells in the TME

To date, most research studies have been based on the role of epigenetic alterations on cancer cells. However, given the relevance of the immune cells in cancer, an increasing number of studies are showing that immune cells in the TME could be regulated by epigenetic modifications. Consequently, the study of the epigenetic alterations that affects immune cell function in the TME has become a growing area of investigation in the last few years.

Epigenetic modifications influence the function of lymphoid cells. DNA methylation by DNMT1 and H3K27 trimethylation by EZH2 have been found to impair T-cell infiltration in the TME [189]. Moreover, the DNA methylation patterns were different in exhausted CD8+ T cells. Thus, the gene LAG3 was methylated in naïve cells and demethylated during the activation of naïve CD8+ T cells [190]. LSD1, a lysine-specific demethylase 1A, also seems to be involved in the infiltration of T cells. Accordingly, the increase in H3K4me2 by LSD1 inhibition allowed more CD8+ T-cells recruitment [191], as shown in breast cancer [192]. Likewise, a DNA hypomethylation pattern is necessary for Treg specific gene expression and its immunosuppressive activity [193]. Furthermore, the pro-inflammatory activity mediated by Tregs in the TME has been associated with EZH2 inhibition [194].

Regarding NK cells, epigenetic alterations have been described to affect their maturation, differentiation, and activation [195]. Specifically, the receptors on the NK cell surface with effector function seem to be regulated epigenetically, which would link the epigenetic with the tumor activity of NK cells.

Different studies have reported an epigenetic regulation by histone acetylation and methylation in activating receptors. Therefore, histone acetylation has been shown to control the NKp30 and NKp46 receptors [196], and the *NKG2D* gene was unmethylated in NK cells and associated with high levels of H3K9 acetylation [197]. Additionally, an increase in the NKG2D receptor has been associated with a decrease in H3K27me3 promoted by an inhibition of EZH2 [74,198]. Initially, a decrease in NK cell activating receptors would be associated with an exhaustion of these cells, which would promote impaired anti-tumor immune responses. However, Yin et al. showed that the expansion and high cytotoxicity of NK cells against malignant cells were associated with an increase in the NKG2D receptor [74].

Since TAMs have been associated with worse prognosis in lymphoma [82,83], a wide variety of studies have tried to elucidate the network that regulates TAM polarization. Epigenetic modifications have described both epigenetic markers that positively regulate M2 polarization and those with the opposite effect. For example, DNMT 3B plays a role in macrophage polarization because its downregulation increases the M2 macrophage expression markers, such as Arg1 [199]. Furthermore, changes in H3K4 and H3K27 methylation seem to control the expression of M2 macrophage markers [200]. Thus, H3K4 methyltransferase SET and SMYD3 (MYND Domain 3) and Jumonji domain containing protein D3 (JMJD3), a H3K27 demethylase, have a role in M2 polarization [200,201,202]. JMJD3 would promote di- and tri-demethylation of H3K27, promoting an activation of Arg1, among other M2 markers [200,202]. Additionally, some HDACs have been associated with the regulation of M2 phenotype, such as HDAC4 and SIRT2, which are implicated in the regulation of *Arg1* expression [203,204]. Concerning epigenetic modifications that negatively regulate M2 polarization, HDAC3 and HDAC9 have been proposed. A knockdown of HDAC3 showed the role of this HDAC in the decrease in inflammation [205]. Furthermore, a depletion of HDAC9 also caused a downregulation of inflammatory genes and accumulation of H3K9 and total acetylated H3 at *ABCA1* (ATP-binding cassette transporter), *ABCG1*, and *PPAR-γ* (peroxisome proliferator-activated receptor) promoters in macrophages [206].

Regarding MDSCs, some studies indicate that HDACs would regulate their immunosuppressive function. Chen et al. described that MDSCs with HDAC11KO were more suppressive within the TME in a murine lymphoma microenvironment. This immunosuppressive activity was associated with the upregulation of expression and enzymatic activity of ARG1 and Nos2 [207]. By contrast, the inhibition of class I histone deacetylases by the epigenetic drug entinostat showed an inhibition of the immunosuppressive function of M-MDSCs. This effect was promoted by the reduction in ARG1, iNOS, and COX-2 levels [208]. Additionally, HDAC11 could be important in the regulation of IL-10 levels in myeloid cells as well as in the expansion of MDSCs [209]. The expression of IL-10 was also reported to potentially be regulated by HDAC6 acting as a transcriptional factor [210]. However, there is no evidence in lymphoma disease. In addition to the role of HDACs, miRNAs may have a role in the regulation of MDSC functions. Thus, a significant increase in miR494 has been identified in MDSCs derived from six different tumor models, at least by using T lymphoma (EG7) and B lymphoma (A20) cell lines. This miRNA promoted the accumulation and activity of MDSCs by Akt pathway activation and targeting the phosphatase and tensin homolog (PTEN) [211].

### 4.3. Epigenetic Cross-Talk between Lymphoma Tumor Cells and TME

Throughout the review, the method by which epigenetics is dysregulated both in lymphoma tumor cells and in cells surrounding the tumor has been described. However, little is known about the epigenetic modifications that participate in the link between lymphoma tumor cells and TME.

**Figure 2:**
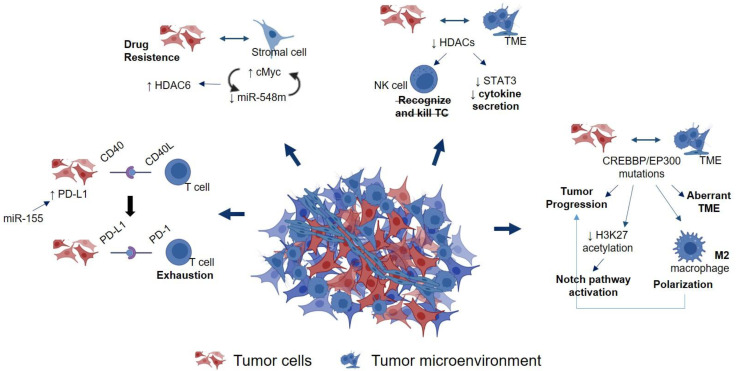
Schematic representation of the epigenetic cross-talk between lymphoma tumor cells (TC) and the tumor microenvironment (TME) in lymphoma diseases.

The reciprocal interplay between tumor cells and the TME components triggers activation of signal cascades that result in specific alterations in gene expression patterns driven by epigenetic mechanisms, as shown in Figure 2. For example, mantle cells lymphoma (MCL) and T cells are able to interact between them due to the expression of CD40 and CD40 ligand inducing PD-L1 on MCL cells [212,213]. PD-L1 expression level is, in part, controlled by epigenetic mechanisms [214]. As a consequence, the increase in PD-L1 on MCL cells enhances PD-L1 and PD1 interactions, which results in T-cell exhaustion and, therefore, the immune escape of lymphoma cells [8,9]. Moreover, recent studies have reported the contribution of micro-RNAs to PD-L1 expression. In DLBCL cells, the miR-155 overexpression increased PD-L1, promoting the immune evasion of lymphoma cells [215] by impairing the cytotoxic CD8+ T cells’ function.

The downregulation of miR-548m was also observed when lymphoma cells and stromal cells interacted with each other. The modification in miR-548m expression caused an alteration of HDAC6 expression. Thereby, there was a balance between miR-548m and HDAC6 to induce lymphoma formation and drug resistance [10].

Furthermore, both HATs and HDACs have been involved in the link between lymphoma tumor cells and the TME. Thus, Irina V. Tiper and Tonya J. Webb hypothesized that malignant cells used epigenetic modifications to dysregulate antigen presentation and to affect the ability of NK cells to recognize and kill cells [216]. They reported that the inhibition of HDACs enhanced the antigen presentation and NK cell responses to MCL cells, and inhibited STAT3 and the inflammatory cytokine secretion by MCL cells. All of this was due to the epigenetic regulation of CD1D by HDAC2, which binds to the CD1D promoter [216].

Regarding HATs, a recent study has shown that CREBBP/EP300 mutations contributed to both tumor progression and an aberrant TME in DLBCL [217]. For this purpose, the authors performed a genomic analysis in a cohort of DLBCL patients, in which it was shown that CREBBP/EP300 mutations were associated with tumor progression. In vitro, CREBBP/EP300 mutations in two B-lymphoma cell lines inhibited H3K27 acetylation and triggered a signaling cascade that resulted in the activation of NOTCH pathway. Moreover, these two mutated B-lymphoma cell lines in mononuclear cells co-cultured with peripheral blood promoted the proliferation of lymphoma cells and the macrophage activation and polarization to their M2 phenotype [217]. Hence, this study demonstrated that epigenetic alterations were implicated in the regulation of both tumor progression and TME in DLBCL.

Despite the studies described above, it is mandatory to obtain a deeper knowledge of the epigenetic cross-talk between lymphoma cells and TME, because tumor progression and drug resistance capacity are guided by the dynamic and mutual communication between tumor cells and TME, in which epigenetics plays a relevant role. Thus, this complex interplay would modify specific epigenetic markers that would be different in the absence of this interaction. Accordingly, this could complicate the use of traditional and epigenetic drugs in the treatment of neoplasms [142].

### 4.4. Epigenetic Therapies: Clinical Trials in Lymphoma Disease

Given the importance of developing novel therapies that can reduce drug resistance and increase the survival rate of lymphoma patients, the epigenetic drugs represent a promising and potentially therapeutic tool.

The first DNMT inhibitors (DNMTi) approved by the Food and Drug Administration (FDA) were azacitidine and decitabine [218]. These epigenetic drugs were used against myelodysplastic syndrome (MDS) and acute myeloid leukemia (AML), with moderate efficacy and high toxicity [219,220]. Moreover, the FDA has approved several epigenetic drugs for the treatment of diverse malignancies including the HDAC inhibitors (HDACi) vorinostat, romidepsin, belinostat, and panobinostat, with vorinostat being the first HDACi approved by the FDA to treat CTCL [221].

Currently, all these epigenetic drugs are being evaluated alone or in combination in several clinical trials involving lymphoma patients. Here, we report the active and recruiting clinical trials in lymphomas which use the previously approved epigenetic drugs (Table 3).

The previous results obtained in clinical trials have shown the effects of these epigenetic drugs in lymphoma patients. Azacitidine has been combined with chemotherapy and epigenetic drugs, among others. In a phase 1/2 trial (NCT01004991), the combination of azacitidine with R-CHOP reported a complete remission (CR) rate of 91.7% in untreated DLBCL patients. Moreover, the combination with vorinostat showed an ORR of 6.7% in patients with relapsed or refractory DLBCL in a phase 1/2 trial (NCT01120834). In B-NHL patients, decitabine decreased tumor DNMT1 and increased tumor apoptosis (*p* < 0.05), mitoses (*p* = 0.02), and copper/platinum transporter CTR1 [222]. Moreover, Blum et al. reported that the dose-limiting myelosuppression and infectious complications prevented dose escalation of decitabine to levels associated with changes in global methylation or gene re-expression in chronic lymphocytic leukaemia and NHL [223].

Regarding HDACis, vorinostat in monotherapy in a phase 1 trial conducted in FL, MCL, DLBL, and CTCL patients (NCT00127140) showed different responses in lymphoma patients, varying from CRs to worse responses [224]. A phase 2 trial (NCT00097929) testing vorinostat presented a CR of 5.6% in relapsed DLBCL. These studies with vorinostat in monotherapy seem to have a limited antitumor activity. However, vorinostat has sustained antitumor activity in patients with relapsed or refractory FL. In this phase 2 study, the ORR was 49% and the median PFS was 20 months for the 39 patients with FL [225]. In a combinatorial treatment, vorinostat plus rituximab in NHLs (phase 2 trial NCT00720876) showed an ORR of 50% in FL, 50% in MZL, 33% in MCL, and no response in LPL [226]. In a phase 1/2 study (NCT00972478), vorinostat combined with R-CHOP presented a tendency to improve R-CHOP efficacy in DLBCL (ORR 81% and CR 52%) [227]. Romidepsin or FK228 reported an ORR of 38% and CR 18% and an ORR of 34% and CR of 5.6% in relapsed or refractory PTCL and CTCL, respectively (phase 2 trial NCT00007345) [228,229]. Another HDACi, belinostat or PXD101, has shown an ORR of 10.5% (CR 0%) in relapsed or refractory aggressive B-NHLs (phase 2 trial NCT00303953) [230], and in another phase 2 trial (NCT00274651) involving relapsed or refractory PTCL or CTCL patients, belinostat presented an ORR of 25% (CR 8.3%) in PTCL and an ORR of 14% (CR 10.3%) in CTCL [231]. Finally, the combination of panobinostat and everolimus in relapsed HL and N-HL patients showed an ORR of 43% and CR of 15% [232].

With the clinical trial results, it might be argued that DNMT and HDAC inhibitors used as monotherapies seem to have limited efficacy in lymphoma in early phase studies. However, clinical trials that used combinatorial regimens have demonstrated potent efficacies in many lymphoma settings with an acceptable level of safety. Therefore, further research is essential to better know and understand the epigenetic mechanisms involved in the progression and prognosis in lymphomas, with the aim of developing new therapeutic approaches that significantly improve the clinical prognosis of patients.

## 5. Conclusions and Future Perspectives

The role of epigenetic alterations in lymphoma and other tumors has been predominantly focused on tumor cells. However, compelling evidence also highlights the relevant role of the TME in supporting the proliferation, migration, and survival of lymphoma cells. For this reason, an increasing number of studies are describing the epigenetic modifications that appear in the TME cells and their role in creating an optimal immunosuppressive environment for tumor development. Nevertheless, a deeper understanding of the interrelationship between lymphoma tumor cells, TME, and the epigenetic mechanisms involved is essential.

The extreme complexity of the interplay between the great variety of cells and the epigenetic alterations occurring in the context of the disease makes the challenge even greater. Nevertheless, a better understanding of this cross-talk would lead to advances in this field and would contribute to increasing our knowledge of the lymphoma disease. Accordingly, the epigenetic alterations could be used as new biomarkers in lymphoma, which would lead to patient stratification based on the expression of the epigenetic marks. Additionally, the use of epigenetic drugs might be an interesting therapeutic option [143] in order to not only address the elimination of lymphoma tumor cells but also counteract the modulating effect of the heterogeneous components that shape the TME. Altogether, it would help to disrupt the dynamic and mutual communication between malignant and immune cells and, consequently, it would contribute to inhibiting tumor progression and chemoresistance or to modifying the immunosuppressive TME activity.

## Figures and Tables

**Table 1:** Immune cells in tumor microenvironment of lymphoproliferative diseases.

Immune Cells in TME	Function	Mechanism	References
Th1 cells	Anti-tumor cells	Produce IL-2, IFN-gamma, and TNF-beta. Activate CTLs, antigen-presenting cells (APCs), and NK cells	[47]
Th2 cells	Pro-tumor cells	Express IL-4, IL-5, IL-6, and IL-10 and support tumor cell growth through CD40–CD40L interactions	[6,48]
Tregs	Pro- and anti-tumor cells	Express co-inhibitory and co-stimulatory markers. Three different Treg subsets have been identified in NHL. High levels of PD-1, OX40, ICOS, TIGIT, CTLA-4 and CD28, CD69, and CD95/Fas (activation markers). The third subset of Tregs had a lack of FOXP3 and a high expression of LAG3 and CTLA-4, IL10, CD38, KLRB1 (immunosuppression-associated genes)	[57]
CTL cells	Pro- and anti-tumor cells	Suppression of the progression of B-cell NHL [42]. Induce the progression in nodal cytotoxic T-cell lymphoma and cutaneous T-cell lymphoma [43,44]	[67,68,69]
B cells	Pro- and anti-tumor	Contribute to immune evasion [48], B cells allowing the progression of the disease inducing anergy in CD4+ T cells by CTLA-4 up-regulation [49]	[7,73]
NK cells	Anti-tumor cells	IL-2 and NKp30 and NKG2D surface receptors	[77]
M2 macrophages	Pro-tumor cells	High number of CD163+/MYC+ cells in HL is associated with worst outcome	[80]
MDSCs	Pro-tumor cells	Inhibit T cell immune responses by reactive oxygen species or nitrogen oxide production. Inhibit the maturation and functions of NK cells and to induce macrophages polarization into M2 phenotype by secreting IL-10 and transforming growth factor (TGF)-β secretion	[93,95,96]

**Table 2:** Epigenetic modifications involved in lymphoma tumors.

Epigenetic Subgroup	Epigenetic Modifications/Mechanism	Function
DNA methylation	DNMT1 overexpression: hipermethylation of tumor supressors promoters/impaired T-cell infiltration in the TME	Pro-tumor cells
	DNMT 3A mutation: hypomethylation and Notch1 pathway activation	Anti-tumor cells (mutation pro-tumor cells)
	DNMT 3B overexpression: hipermethylation MYC promoter; downregulation: increases M2 macrophage expression markers (*Arg1*)	Pro-tumor cells
Histone acetylation	HDAC1 overexpression: inhibits the tumor supressor BIM and increase in IL-15 secretion	Pro-tumor cells
	HDAC2 and EZH2 overexpression: inhibits the tumor supressor BIM/mpaired T-cell infiltration in the TME	Pro-tumor cells
	HDAC3 overexpression: downregulation: downregulation of inflammatory genes	Pro-tumor cells (downregulation anti-tumor cells)
	HDAC6 overexpression: increase in IL-15 secretion	Pro-tumor cells (PTCL) and anti-tumor cells (DLBCL)
	HDAC9 overexpression: modulates BCL6 activity and inhibits the tumor supressor p53; downregulation: downregulation of inflammatory genes	Pro-tumor cells (downregulation pro-tumor cells)
	HDAC11: impaires immunosuppressive–MDSC capacity	Anti-tumor cells
Histone methylation	EZH2 (catalyzes H3K27me3) overexpression: regulates MYC and induces high proliferation of/increases in NKG2D receptor	Pro-tumor cells
	JMJD3 (promotes di- and tri-demethylation of H3K27) induces activation of Arg1, among other M2 markers	Pro-tumor cells
	LSD1 (mono- or di-demethylase for H3K4) regulates infiltration of CD8+ T-cells into tumors	Anti-tumor cells (downregulation pro-tumor cells)
	MLL2 (catalyzes H3K4 monomethyltransferase) mutation: alters genes expression (ARID1A, TRAF3) or signaling pathways (JAK-STAT or MAPK)	Anti-tumor cells (mutation pro-tumor cells)
	SETD2 (catalyzes H3K36me3) mutation: enteropathy-associated T-cell lymphoma	Anti-tumor cells (mutation pro-tumor cells)
	SMYD3 (catalyzes H3K4 and H4K5 methyltransferase) induces activation of Arg1, among other M2 markers	Pro-tumor cells
Non-coding RNA	circ-APC regulates tumor cell proliferation	Anti-tumor cells (downregulation pro-tumor cells)
	let-7 family inhibits MYC expression	Anti-tumor cells
	miR-34b inhibits MYC expression	Anti-tumor cells
	miR494 promotes the accumulation and activity of MDSCs by Akt pathway activation	Pro-tumor cells
	miRNA-21 downregulation: increases PTEN expression and induces apoptosis	Anti-tumor cells

**Table 3:** Clinical trials of FDA-approved epigenetic drugs in lymphoproliferative diseases.

NCT Number	Title	Conditions	Phase
SAHA/Vorinostat (HDACi)			
NCT00972478	Vorinostat, Rituximab, and Combination Chemotherapy in Treating Patients With Newly Diagnosed Stage II, Stage III, or Stage IV Diffuse Large B-Cell Lymphoma	Ann Arbor Stage II Non-Hodgkin Lymphoma Ann Arbor Stage III Non-Hodgkin Lymphoma Ann Arbor Stage IV Non-Hodgkin Lymphoma	Phase 1 Phase 2
NCT00336063	Vorinostat and Azacitidine in Treating Patients with Locally Recurrent or Metastatic Nasopharyngeal Cancer or Nasal Natural Killer T-Cell Lymphoma	Adult Nasal Type Extranodal NK/T-Cell Lymphoma Recurrent Nasopharyngeal Keratinizing Squamous Cell Carcinoma Recurrent Nasopharyngeal Undifferentiated Carcinoma Stage IV Nasopharyngeal Keratinizing Squamous Cell Carcinoma AJCC v7 Stage IV Nasopharyngeal Undifferentiated Carcinoma AJCC v7	Phase 1
NCT03150329	Pembrolizumab and Vorinostat in Treating Patients with Relapsed or Refractory Diffuse Large B-Cell Lymphoma, Follicular Lymphoma, or Hodgkin Lymphoma	Grade 3b Follicular Lymphoma Recurrent B-Cell Lymphoma, Unclassifiable, with Features Intermediate Between Diffuse Large B- Cell Lymphoma and Classic Hodgkin Lymphoma Recurrent Classic Hodgkin Lymphoma Recurrent Diffuse Large B-Cell Lymphoma Recurrent Follicular Lymphoma Recurrent Grade 1 Follicular Lymphoma Recurrent Grade 2 Follicular Lymphoma Recurrent Grade 3a Follicular Lymphoma Recurrent Primary Mediastinal (Thymic) Large B-Cell Cell Lymphoma Recurrent Transformed Non-Hodgkin Lymphoma	Phase 1
NCT01193842	Vorinostat and Combination Chemotherapy with Rituximab in Treating Patients With HIV-Related Diffuse Large B-Cell Non-Hodgkin Lymphoma or Other Aggressive B-Cell Lymphomas	AIDS-Related Plasmablastic Lymphoma AIDS-Related Primary Effusion Lymphoma Ann Arbor Stage I Diffuse Large B-Cell Lymphoma Ann Arbor Stage I Grade 3 Follicular Lymphoma Ann Arbor Stage II Diffuse Large B-Cell Lymphoma Ann Arbor Stage II Grade 3 Contiguous Follicular Lymphoma Ann Arbor Stage II Grade 3 Non- Contiguous Follicular Lymphoma Ann Arbor Stage III Diffuse Large B-Cell Lymphoma Ann Arbor Stage III Grade 3 Follicular Lymphoma Ann Arbor Stage IV Diffuse Large B-Cell Lymphoma	Phase 1 Phase 2
Romidepsin (HDACi)			
NCT03547700	Study of Ixazomib and Romidepsin in Peripheral T-cellLymphoma (PTCL)	Lymphoma, T-Cell, Peripheral	Phase 1 Phase 2
NCT03141203	Evaluation of the Combination of Romidepsin and Carfilzomib in Relapsed/Refractory Peripheral T Cell Lymphoma Patients	Peripheral T-Cell Lymphoma	Phase 1 Phase 2
NCT01796002	Efficacy and Safety of Romidepsin CHOP vs. CHOP inPatients with Untreated Peripheral T-Cell Lymphoma	Peripheral T-Cell Lymphoma	Phase 3
NCT02616965	A Study to Assess the Feasibility of Romidepsin Combined with Brentuximab Vedotin in Cutaneous T-cell Lymphoma	Cutaneous T-Cell Lymphoma (CTCL)	Phase 1
NCT01908777	A Phase 2 Multicenter Study of High Dose Chemotherapy with Autologous Stem Cell Transplant Followed by Maintenance Therapy with Romidepsin for the Treatment of T-Cell Non-Hodgkin Lymphoma	T-Cell Non-Hodgkin Lymphoma	Phase 2
NCT01755975	Romidepsin in Combination with Lenalidomide in Adultswith Relapsed or Refractory Lymphomas and Myeloma	Multiple Myeloma Non-Hodgkin’s Lymphoma	Phase 1 Phase 2
NCT03534180	Venetoclax and Romidepsin in Treating Patients withRecurrent or Refractory Mature T-Cell Lymphoma	Anaplastic Large Cell Lymphoma Recurrent Mature T-Cell and NK-Cell Non-Hodgkin Lymphoma Refractory Mature T-Cell and NK-Cell Non-Hodgkin Lymphoma	Phase 2
Belinostat (HDACi)			
NCT02737046	Belinostat Therapy With Zidovudine for Adult T-CellLeukemia-Lymphoma	Adult T-Cell Leukemia–Lymphoma ATLL	Phase 2
Panobinostat (HDACi)			
NCT01261247	Panobinostat in Treating Patients with Relapsed orRefractory Non-Hodgkin Lymphoma	Adult Nasal Type Extranodal NK/T-Cell Lymphoma Anaplastic Large Cell Lymphoma Angioimmunoblastic T-Cell Lymphoma Extranodal Marginal Zone B-Cell Lymphoma of Mucosa-associated Lymphoid Tissue Hepatosplenic T-Cell Lymphoma Nodal Marginal Zone B-Cell Lymphoma Peripheral T-Cell Lymphoma Post-transplant Lymphoproliferative Disorder Recurrent Adult Burkitt Lymphoma Recurrent Adult Diffuse Large Cell Lymphoma	Phase 2
5-azacytidine (DNMTi)			
NCT03450343	Oral Azacitidine Plus Salvage Chemotherapy inRelapsed/Refractory Diffuse Large B-Cell Lymphoma	Large B-Cell Diffuse Lymphoma	Phase 1
NCT03703375	Efficacy and Safety of Oral Azacitidine (CC-486) Compared to Investigator’s Choice Therapy in Patients with Relapsed or Refractory Angioimmunoblastic T-Cell Lymphoma	Lymphoma, T-Cell	Phase 3
NCT04897477	Azacytidine, Bendamustine, Piamprizumab inRefractory/Relapsed B-Cell Non-Hodgkin Lymphoma	Non-Hodgkin Lymphoma, B-Cell	Phase 1 Phase 2
NCT04480125	Azacitidine Combined with Chidamide in the Treatment of Newly Diagnosed PTCL Unfit for Conventional Chemotherapy	Peripheral T-Cell Lymphoma	Phase 2
NCT03593018	Efficacy and Safety of Oral Azacitidine Compared to Investigator’s Choice Therapy in Patients with Relapsed or Refractory AITL	Relapsed Angioimmunoblastic T-Cell Lymphoma Refractory Angioimmunoblastic T-Cell Lymphoma	Phase 3
NCT04747236	A Randomized, Phase IIB, Multicenter, Trial of Oral Azacytidine Plus Romidepsin Versus Investigator’s Choice in Patients with Relapse or Refractory Peripheral T-Cell Lymphoma (PTCL)	PTCL	Phase 2
NCT05162976	CC-486 and Nivolumab for the Treatment of HodgkinLymphoma Refractory to PD-1 Therapy or Relapsed	Recurrent Classic Hodgkin Lymphoma Refractory Classic Hodgkin Lymphoma	Phase 1
NCT03542266	CC486-CHOP in Patients with Previously UntreatedPeripheral T-Cell Lymphoma	Previously Untreated Peripheral T-Cell Lymphoma	Phase 2
NCT04578600	CC-486, Lenalidomide, and Obinutuzumab for the Treatment of Recurrent or Refractory CD20 Positive B- Cell Lymphoma	Indolent B-Cell Non-Hodgkin Lymphoma Recurrent B-Cell Non-Hodgkin Lymphoma Recurrent Chronic Lymphocytic Leukemia Recurrent Mucosa-associated Lymphoid Tissue Lymphoma Recurrent Follicular LymphomaRecurrent Hairy Cell Leukemia Recurrent Lymphoplasmacytic Lymphoma Recurrent Mantle Cell Lymphoma Recurrent Marginal Zone Lymphoma Refractory B-Cell Non-Hodgkin Lymphoma	Phase 1
NCT02828358	Azacitidine and Combination Chemotherapy in Treating Infants With Acute Lymphoblastic Leukemia and KMT2A Gene Rearrangement	Acute Leukemia of Ambiguous Lineage B Acute Lymphoblastic Leukemia Mixed Phenotype Acute Leukemia	Phase 2
5-Aza-2-deoxycytidine (DNMTi)			
NCT02951728	Decitabine Plus R-CHOP in Diffuse Large B-CellLymphoma	Diffuse Large B-Cell Lymphoma	Phase 1 Phase 2
NCT04697940	Decitabine-primed Tandem CD19/CD20 CAR T-Cells’Treatment in r/r B-NHL	Relapase and Refractory B-Cell Non- Hodgkin Lymphoma Decitabine-primed Tandem CD19/CD20 CAR T Cells	Phase 1 Phase 2
NCT04850560	Sequential Low-dose Decitabine with PD-1/CD28 CD19CAR-T in Relapsed or Refractory B-Cell Lymphoma	Objective Response Rate	Phase 1
NCT03494296	A Prospective Study of Low-dose Decitabine Combined with COP Regimen in the Treatment of Relapsed and Refractory DLBCL	Lymphoma	
NCT04446130	Study of Decitabine Combined with HAAG Regimen in Newly Diagnosed ETP-ALL/LBL, T/M-MPAL, and ALL/ LBL with Myeloid or Stem Cell Markers Patients	Induction Chemotherapy Acute T-Lymphocytic Leukemia T-Cell Lymphoblastic Lymphoma Leukemia T-Cell/Myeloid Mixed Phenotype Acute Leukemia	Phase 3
NCT04553393	Decitabine-primed Tandem CD19/CD20 CAR T-Cells Plus Epigenetic Agents in Aggressive r/r B-NHL with Huge Tumor Burden	Refractory or Relapsed Aggressive r/r B- NHL With Huge Tumor Burden	Phase 1 Phase 2
